# Automatic Extraction of Blood Vessels in the Retinal Vascular Tree Using Multiscale Medialness

**DOI:** 10.1155/2015/519024

**Published:** 2015-04-22

**Authors:** Mariem Ben Abdallah, Jihene Malek, Ahmad Taher Azar, Philippe Montesinos, Hafedh Belmabrouk, Julio Esclarín Monreal, Karl Krissian

**Affiliations:** ^1^Faculty of Sciences, Electronics and Microelectronics Laboratory, Monastir University, 5019 Monastir, Tunisia; ^2^Faculty of Computers and Information, Benha University, Benha 13511, Egypt; ^3^Institute of Mines and Ales, Laboratory of Computer and Production Engineering, 30319 Alès, France; ^4^Imaging Technology Center (CTIM), Las Palmas-Gran Canaria University, 35017 Las Palmas de Gran Canaria, Spain

## Abstract

We propose an algorithm for vessel extraction in retinal images. The first step consists of applying anisotropic diffusion filtering in the initial vessel network in order to restore disconnected vessel lines and eliminate noisy lines. In the second step, a multiscale line-tracking procedure allows detecting all vessels having similar dimensions at a chosen scale. Computing the individual image maps requires different steps. First, a number of points are preselected using the eigenvalues of the Hessian matrix. These points are expected to be near to a vessel axis. Then, for each preselected point, the response map is computed from gradient information of the image at the current scale. Finally, the multiscale image map is derived after combining the individual image maps at different scales (sizes). Two publicly available datasets have been used to test the performance of the suggested method. The main dataset is the STARE project's dataset and the second one is the DRIVE dataset. The experimental results, applied on the STARE dataset, show a maximum accuracy average of around 94.02%. Also, when performed on the DRIVE database, the maximum accuracy average reaches 91.55%.

## 1. Introduction

**Figure 1:**
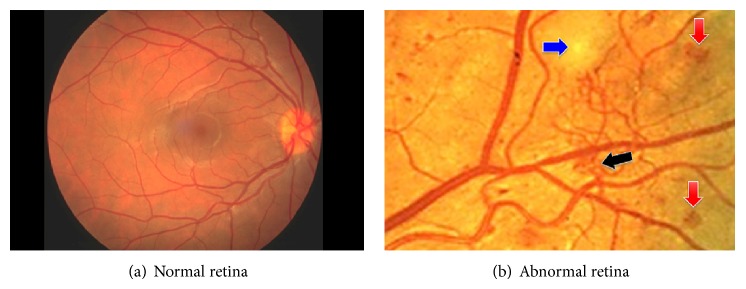
Retinal images [32].

For decades, retinal images are widely used by ophthalmologists for the detection and follow-up of several pathological states [1–5]. Fundus photographs, also called retinal photography, are captured using special devices called “Charged Coupled Devices” (CCD), which are cameras that show the interior surface of the eye [6–10]. These images directly provide information about the normal and abnormal features in the retina. The normal features include the optic disk, fovea, and vascular network. There are different kinds of abnormal features caused by diabetic retinopathy (DR) such as microaneurysm, hard exudate, soft exudate, hemorrhage, and neovascularization. An example of retinal images obtained by fundus photography is given in Figure 1, where two retinal images are shown. The first one does not show any DR sign (Figure 1(a)) and the second one demonstrates advanced-DR signs indicated by color arrows (Figure 1(b)). However, the manual detection of blood vessels is very difficult since the blood vessels in these images are complex and have low level contrast [11]. Also, not all the images show signs of diabetic retinopathy. Hence, a manual measurement of the information about blood vessels, such as length, width, tortuosity, and branching pattern, becomes tedious. As a result, it increases the time of diagnosis and decreases the efficiency of ophthalmologists. Therefore, automatic methods for extracting and measuring the vessels in retinal images are needed to save the workload of the ophthalmologists and to assist in characterizing the detected lesions and identifying the false positives [12].

Several works have been proposed for detecting the 2D complex vessel network, such as single scale matched filter [13–15], multiscale matched filter [16], adaptive local thresholding [17], single-scale Gabor filters [18], and multiscale Gabor filters [19]. Cinsdikici and Aydin [20] put forward a blood vessel segmentation based on a novel hybrid model of the matched filter and the colony algorithm, which extracts vessels perfectly but the pathological areas can affect the result. In [21–23] authors adapted another approach which applied mathematical morphological operators. The suggested method in [21] proved to be a valuable tool for the segmentation of the vascular network in retinal images, where it allowed obtaining a final image with the segmented vessels by iteratively combining the centerline image with the set of images that resulted from the vessel segments' reconstruction phase using the morphological operator. However, the inconvenience of this method is when a vessel centerline is missing, so the corresponding segmented vessel is normally not included in the final segmentation result. In [22], the authors proved that it was possible to select vessels using shape properties and connectivity, as well as differential properties like curvature. The robustness of the algorithm has been evaluated and tested on eye fundus images and on other images. Gang et al. [24] showed that the Gaussian curve is suitable for modeling the intensity profile of the cross section of the retinal vessels in color fundus images. Based on this elaboration, they proposed the amplitude-modified second-order Gaussian filter for retinal vessel detection, which optimized the matched filter and improved the successfulness of the detection. Staal et al. [25] explained a method for an automated segmentation of vessels in two-dimensional color images. The system was based on extracting image ridges that coincide approximately with vessel centerlines, where the evaluation was done using the accuracy of hard classifications and the values of soft ones. In [26], the authors presented a hybrid method for an efficient segmentation of multiple oriented blood vessels in colour retinal images. The robustness and accuracy of the method demonstrated that it might be useful in a wide range of retinal images even with the presence of lesions in the abnormal images. Dua et al. [27] presented a method for detecting blood vessels, which employs a hierarchical decomposition based on a quad tree decomposition. The algorithm was faster than the existing approaches. In the recent years, alternative approaches for an automated vessel segmentation have used the Hessian-based multiscale detection of curvilinear structures, which has been effective in discerning both large and small vessels [28–31].

In this paper, we propose a multiscale response to detect linear structures in 2D images. We will use the formulation, which was suggested in [36, 37]. The presented detection algorithm is divided into two steps. First, we present a flux-based anisotropic diffusion method and apply it to denoise images corrupted by an additive Gaussian noise. In order to extract only the pixels belonging to a vessel region, we use a Gaussian model of the vessels for interpreting the eigenvalues and the eigenvectors of the Hessian matrix. Then, we compute the multiscale response from responses computed at a discrete set of scales. The method has been evaluated using the images of two publicly available databases, the DRIVE database [34] and the STARE database [33]. Prior to analysing fundus images, we have used the green channel alone, since it gives the highest contrast between the vessel and the background.

## 2. Methodology

### 2.1. Preprocessing Technique

In the ocular fundus image, edges and local details between heterogeneous regions are the most interesting part for clinicians. Therefore, it is very important to preserve and enhance edges and local fine structures and simultaneously reduce the noise. To reduce the image noise, several approaches have been proposed using techniques such as linear and nonlinear filtering. In linear spatial filtering, such as Gaussian filtering, the content of a pixel is given by the value of the weighted average of its immediate neighbors. This filtering not only reduces the amplitude of noise fluctuations but also degrades sharp details such as lines or edges, so the resulting images appear blurred and diffused [24, 38]. This undesirable effect can be reduced or avoided by designing nonlinear filters. The most common technique is median filtering. With it the value of an output pixel is determined by the median of the neighborhood pixels. This filtering retains edges but results in a loss of resolution by suppressing fine details [39]. In order to perform this task, Perona and Malik (PM) [18] developed an anisotropic diffusion method, a multiscale smoothing, and the edge detection scheme, which were a powerful concept in image processing. The anisotropic diffusion was inspired from the heat diffusion equation by introducing a diffusion function, *g*, which depended upon the norm of the gradient of the image:

(1) $$\begin{array}{c} \frac{\partial u}{\partial t}=\mathrm{div}\left(g\left(\left|\nabla u\right|\right)\cdot \nabla u\right), \end{array}$$

where ∇ and *u*(*x*, *t*) denote gradient operation and image intensity, respectively, div is the divergence operator, and |·| denotes the magnitude. The variable *x* represents the spatial coordinate, while the variable *t* is used to enumerate iteration steps in the discrete implementation. Perona and Malik suggested the following diffusion functions:

(2) $$\begin{array}{c} g\left(\left|\nabla u\right|\right)=\frac{1}{1+{\left(\left|\nabla u\right|/k\right)}^{2}},\\ g\left(\left|\nabla u\right|\right)=\exp\left[-{\left(\frac{\left|\nabla u\right|}{k}\right)}^{2}\right], \end{array}$$

where *k* is a parameter of the norm gradient. In this method of anisotropic diffusion, the norm gradient is used to detect edges or frontiers in the image as a step of intensity discontinuity. To understand the relation between the parameter *k* and the discontinuity value |∇*u*|, *F*(∇*u*) can be defined as the following product *F*(∇*u*) = *g* × ∇*u*, called the flow diffusion.

- If |∇*u* | ≫*k*, then *g*(|∇*u*|) → 0 and we have a filter pass-all.
- If |∇*u* | ≪*k*, then *g*(|∇*u*|) → 1 and we obtain an isotropic diffusion filter (like a Gaussian filter), which is a low-pass filter that attenuates high frequencies.

The one-dimensional discrete implementation of (1) is given by

(3) ∂u∂tx,t =∂∂x(gx,t·∇(u)(x,t)) ≈∂∂xgx,t·1dxu(x+dx2,t)−u(x−dx2,t) ≈1dx2gx+dx2,t·(u(x+dx,t)−u(x,t))     −gx−dx2,t·ux,t−ux−dx,t ≈Fright−Fleft if  dx=1,

where *F*_right_ = *F*(*x* + (*dx*/2), *t*) and *F*_left_ = *F*(*x* − (*dx*/2), *t*).

The above result is generalized in *n*-dimensional:

(4) $$\begin{array}{c} \frac{\partial u}{\partial t}\approx \overset{n}{\underset{i=1}{\sum }}F_{x_{i}^{+}}-F_{x_{i}^{-}} \end{array}$$

if ∀*i*, *dx*_*i*_ = 1, *F*_*x*_*i*_^+^_ = *F*_*x*_*i*__(*x* + (*dx*_*i*_/2), *t*) and *F*_*x*_*i*_^−^_ = *F*_*x*_*i*__(*x* − (*dx*_*i*_/2), *t*).

**Figure 2:**
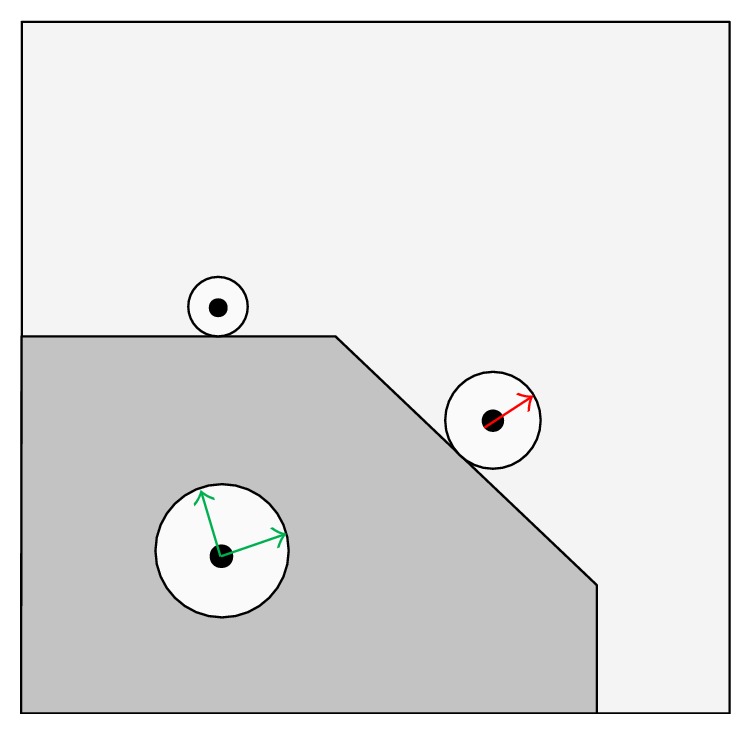
PM anisotropic diffusion.

**Figure 3:**
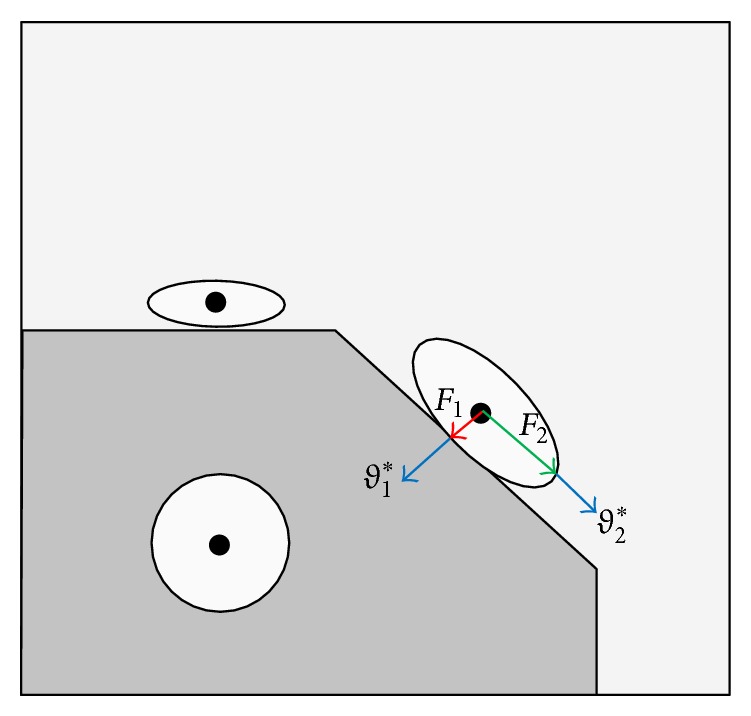
Directional anisotropic diffusion.

Up to now, the anisotropic diffusion has been defined as the case where the diffusivity is a scalar function varying with the location in the image. As described earlier, the PM diffusion (Figure 2) limits the smoothing of an image near the pixels with a high gradient magnitude (edge pixels). As the diffusion near an edge is very weak, the noise smoothing near the edge is also small. To address this, diffusions using matrices instead of scalars have been put forward [36, 40, 41], where the anisotropic diffusion allows the diffusion to be different along various directions defined by the local geometry of the structures in the image (Figure 3). Thus, the diffusion on both sides of an edge can be prevented while allowing the diffusion along the edge. This prevents the edge from being smoothed and then being removed during denoising.

The *F* flux of the matrix diffusion (MD) form can be written as

(5) $$\begin{array}{c} \mathrm{div}\left(D\nabla u\right), \end{array}$$

where *D* is a positive definite symmetrie matrix that may be adapted to the local image structure, which can be written in terms of its eigenvectors *v*_1_ and *v*_2_ and eigenvalues *λ*_1_ and *λ*_2_, as follows:

(6) $$\begin{array}{c} D=\left[\begin{array}{cc} v_{1} & v_{2} \end{array}\right]\left[\begin{array}{cc} \lambda_{1} & 0\\ 0 & \lambda_{2} \end{array}\right]\left[\begin{array}{c} v_{1}^{T}\\ v_{2}^{T} \end{array}\right]. \end{array}$$

Subsequently, the gradient vector field can be written as

(7) $$\begin{array}{c} \nabla u=u_{v_{1}}v_{1}+u_{v_{2}}v_{2}. \end{array}$$

Following the eigenvalues and eigenvectors that we have chosen, different matrix diffusions can be obtained [36, 41]. The diffusion matrix proposed by Weickert et al. [41] had the same eigenvectors as the structure tensor, with eigenvalues that are a function of the norm of the gradient [41, 42]. In our work, we have used a 2D basis (*v*_1_^∗^, *v*_2_^∗^) which corresponds, respectively, to unit vectors in the directions of the gradient and to the minimal curvature of the regularized (or smoothed) version of the image, which is the image convolved with a Gaussian filter with a standard deviation *σ*. This basis is of particular interest in the context of small, elongated structures such as blood vessels, where the minimal curvature holds for the axis direction orthogonal to the gradient. These directions are obtained as two of the eigenvectors of the Hessian matrix of the smoothed image: *H*_*σ*_ (further details are described in Section 2.3). Therefore, the eigenvectors are defined as follows:

(8) $$\begin{array}{c} v_{1}^{*}||\nabla u_{\sigma },\\ v_{2}^{*}⊥\nabla u_{\sigma }, \end{array}$$

where ∇*u*_*σ*_ is the gradient of the image convolved with a Gaussian filter with a standard deviation *σ*, *v*_2_^∗^ gives an estimation of the vessel direction, and *v*_1_^∗^ is its orthogonal. Also, we have used the eigenvalues in (6) as a diffusion function associated to each vector of the basis depending on the first order derivative of the intensity in this direction, instead of the traditional norm of the smoothed gradient. Furthermore, the diffusion can be decomposed as a sum of diffusions in each direction of the orthogonal basis and the divergence term can be written as [36]

(9) $$\begin{array}{c} \mathrm{div}\left(F\right)=\mathrm{div}\left(\overset{2}{\underset{i=1}{\sum }}\phi_{i}\left(u_{v_{i}^{*}}\right)\cdot v_{i}^{*}\right)=\overset{2}{\underset{i=1}{\sum }}\mathrm{div}\left(\phi_{i}\left(u_{v_{i}^{*}}\right)\cdot v_{i}^{*}\right), \end{array}$$

where *u*_*v*_*i*_^∗^_ and *ϕ*_*i*_ indicate the first order derivative of the intensity in the direction *v*_*i*_ and the *i*th diffusion function, respectively. Also, *ϕ*_1_ can be chosen to be any of the diffusivity functions from the traditional nonhomogeneous isotropic diffusion equation, which depends on the first order derivative of the intensity in this direction, as *ϕ*_1_(*u*_*v*_1_^∗^_) = *u*_*v*_1_^∗^_ *e* ^−(*u*_*v*_1_^∗^_/*k*)^2^^ and *ϕ*_2_(*u*_*v*_2_^∗^_) = *α* · *u*_*v*_2_^∗^_, with 0 < *α* < 1, being only a diffusing function to allow smoothing in a *v*_2_^∗^ direction. For further details, the reader could refer to [36, 43].

As in [36], we use a data attachment term with a coefficient *β* which allows a better control of the extent to which the restored image differs from the original image *u*_0_ (at *t* = 0) and of the result of the diffusion process at convergence. The anisotropic diffusion equation becomes

(10) $$\begin{array}{c} \frac{\partial u}{\partial t}=\overset{2}{\underset{i=1}{\sum }}\mathrm{div}\left(\phi_{i}\left(u_{v_{i}^{*}}\right)\cdot v_{i}^{*}\right)+\beta \left(u-u_{0}\right). \end{array}$$

**Figure 4:**
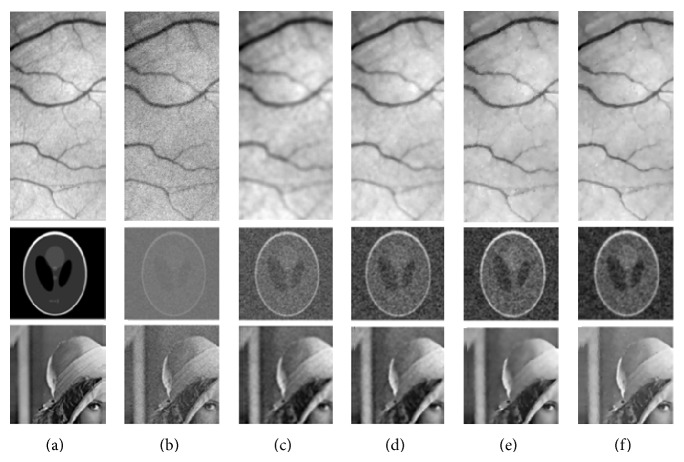
Original images (a) and the corresponding images with additive Gaussian noise (b); denoised images: best result with GF (c), best result with MF (d), best result with PM filter (e), and best result with directional anisotropic diffusion filter (f).

In order to evaluate the denoising effects of the directional anisotropic diffusion (DAD), we have added a Gaussian white noise to each of the images in Figure 4. Once the diffusion method is applied to these noisy images, its effectiveness in reducing the noise is got by calculating the peak signal to noise ratio (PSNR) relative to the original image as follows:

(11) $$\begin{array}{c} \text{PSNR}=10\cdot \log_{10}\left(\frac{d^{2}}{\text{MSE}}\right), \end{array}$$

where *d* = 255 and MSE is the mean-squared error which is written as

(12) $$\begin{array}{c} \text{MSE}=\frac{1}{NM}\overset{N}{\underset{i=1}{\sum }}\overset{M}{\underset{j=1}{\sum }}{\left(I_{\text{original}}\left(i,j\right)-I_{\text{denoised}}\left(i,j\right)\right)}^{2}, \end{array}$$

where *I*_original_ refers to the original image without noise and *I*_denoised_ is the image after the denoising process.

The higher the PSNR is, the better the effect of the denoising is. Note that this measure does not necessarily imply that an image with a higher PSNR is also more visually gratifying. However, based on our experiments using the three test images with an additive white Gaussian noise, we can draw some observations. First, all the techniques we have tried have several parameters that must be selected carefully to obtain the best results. Since we have a “clean” original image, as well as one with noise, we can use the increment in the PSNR value to guide our choice of the parameters. These parameters and the obtained results are indicated in Tables 1, 2, and 3, where we can observe that for the images corrupted with an additive Gaussian noise, the DAD method performs better than the PM method. It gains a higher PSNR (40.4337, 20.9045, and 33.3515) and a smaller MSE (5.8845, 527.9932, and 30.0557) than the aforementioned three methods.

**Figure 5:**
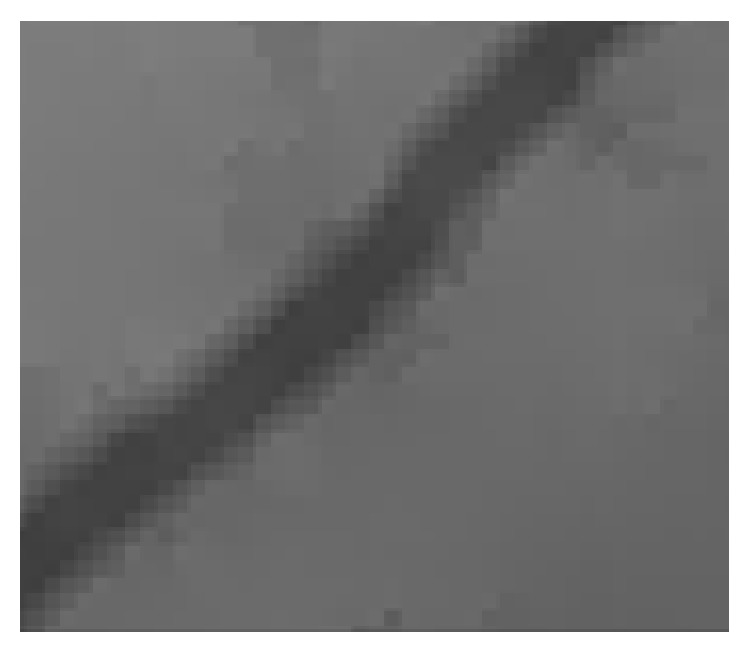
PM anisotropic diffusion (*k* = 3, *N* = 100).

**Figure 6:**
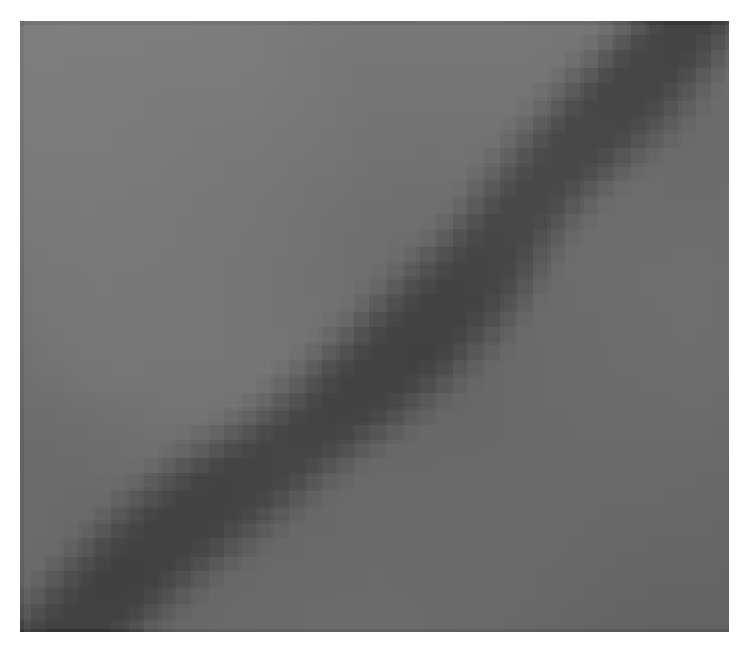
Directional anisotropic diffusion (*k* = 3, *N* = 100, *α* = 0.5).

Figure 4 represents some of the best results for the different methods (GF, MF, PM, and DAD) on the presented three test images (Vessels, phantom, and Lena). For instance, the results recorded after applying the DAD method show that this latter improves much more the visual rendering of the image compared to other methods. As shown in the images of the first row, a DAD filter can effectively improves the quality of a noisy image and also well enhances edges and preserves more details than other filters. Indeed, the Gaussian filter smooths very strongly the planar areas which causes loss of information regarding the fine structures of the image, and it blurs the image. The Median filter, compared to the Gaussian filter, preserves edges but losses details. Comparing the results of the DAD method to those obtained by the PM diffusion in Figures 5 and 6, we can derive several observations. The denoising of PM diffusion model is sensitive to the value of the conductance parameter *k*, and, therefore, smoothing is performed along ridges but not across a ridge line which causes enhancing the desired ridges as well as the noise. To be compared to the DAD diffusion filter, the diffusivity is a tensor-valued function varying with the location and orientation of edges in an image. So, when this filter is applied to a ridge line smoothing is performed along ridges as across a ridge line while preserving the details.

### 2.2. Multiscale Medialness

**Figure 7:**
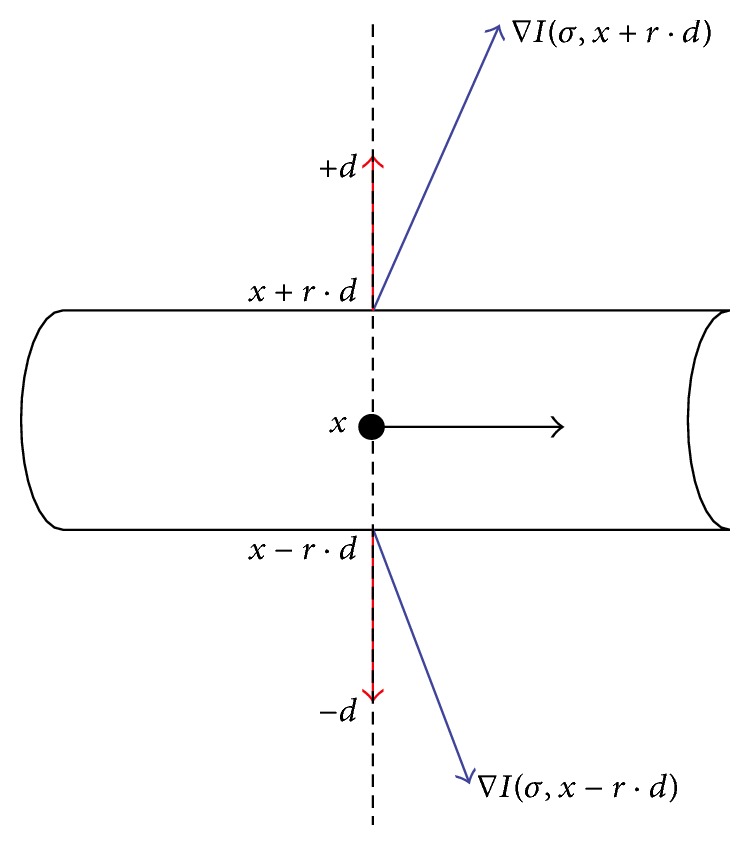
Representation of vesselness measure calculation (from the point *x* on the central line, *d* is the unit vector perpendicular to the main direction of the vessel and *r* = *σ* is the current scale).

The general approach of multiscale methods is to choose a range of scales between *t*_min⁡_ and *t*_max⁡_ (corresponding to *σ*_min⁡_ and *σ*_max⁡_), which are discretized using a logarithmic scale in order to have more accuracy for low scales and to compute a response for each scale from the initial image [36, 43, 47]. The user specifies the minimal and maximal radius of the vessels to extract. Thus, the computation of the single scale response requires different steps. First, a number of points are preselected using the eigenvalues of the Hessian matrix. These points are expected to be near a vessel axis. Then, for each preselected point, the response is computed at the current scale *σ*. The response function uses eigenvectors of the Hessian matrix of the image to define at each point an orientation *D*(*σ*, *x*) orthogonal to the axis of a potential vessel that goes through *M*. From this direction, the two points located at an equal distance of *M* in the direction *D*, noted *M*_1_ and *M*_2_ (Figure 7). The response *R*_*σ*_(*I*) at *M* is taken as the maximum absolute value, among these two points, of the first derivative of the intensity in the *D* direction:

(13) Rσ(x) =max⁡⁡∇σIσ,x+σ·d·+d,∇σIσ,x−σ·d·(−d),

where *d* is the unitary vector of the direction *D*, that is, $d=\vec{v_{1}}$, and ∇_*σ*_ *I* is the gradient of the image at the scale *σ*. ∇_*σ*_ *I* is obtained by the convolution with the first derivative of a Gaussian function of the standard deviation *σ*, where multiplying the derivatives by *σ* ensures the scale invariance property and allows comparing the responses obtained from different scales. The gradient vector ∇_*σ*_ *I* can be computed by a bilinear interpolation for better accuracy, which is especially needed when looking at small vessels [37, 39].

A vessel of a radius *r* is detected at a scale *t*, so we use the scales corresponding to each radius for the multiscale processing. For a fixed scale *t*, we calculate a response image *R*_*t*_(*I*) where *I* is the initial image. Then we calculate the multiscale response for the image *R*_multi_(*I*) which is the maximum of the responses over scales: for each point *x* ∈ *I* and a range [*t*_min⁡_, *t*_max⁡_] of scale:

(14) $$\begin{array}{c} R_{\text{multi}}\left(x\right)=\underset{t}{\max}\left\{R_{t}\left(x\right),\;\;t\in \left[t_{\min},t_{\max}\right]\right\}. \end{array}$$

This response *R*_multi_(*x*) can be interpreted as an indicator that the point *x* belongs to the center line of a vessel, and *R*_*t*_(*x*) can be interpreted as an indicator that the point *x* belongs to the center line of a vessel with a radius *t*. Finally, this response is normalized to give a multiscale response that combines interesting features of each single scale response.

One difficulty with the multiscale approach is that we want to compare the result of a response function at different scales, whereas the intensity and its derivatives are decreasing scale functions. So far, all considerations have been made at a single scale defined by the scale parameter *σ*. In his work, about scale space theory, Lindeberg and Fagerström [48] showed the need for a multiscale analysis to take the varying size of objects into account. He also showed the necessity of normalizing the spatial derivatives between different scales. Thus, the normalized vesselness response is obtained by the product of the normalization term *σ* ^*γ*^ and the final vesselness:

(15) $$\begin{array}{c} R^{*}\left(\Sigma ,\gamma ,x\right)∶=\underset{\sigma \in \Sigma }{\max}\sigma^{\gamma }\cdot R\left(\sigma ,x\right)=\underset{i=1,\ldots ,n}{\max}\sigma_{i}^{\gamma }\cdot R\left(\sigma_{i},x\right). \end{array}$$

**Figure 8:**
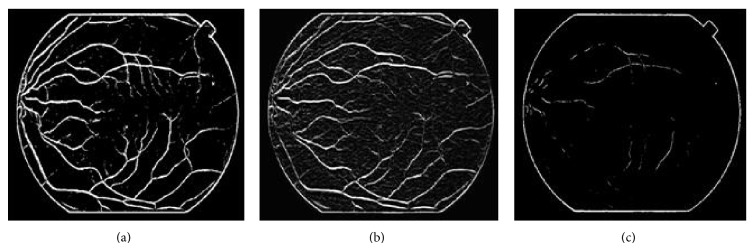
Influence of the normalization parameter *γ* on multiscale response; (a) *γ* = 1 is neutral; (b) *γ* > 1 favors large scales; finally, (c) *γ* < 1 favors small scales.

The parameter *γ* can be used to indicate the preference for a particular scale (Figure 8). If it is set to one, no scale is preferred. Besides, the multiscale response is got by selecting the maximum response over a set of different scales between *σ*_min⁡_ and *σ*_max⁡_.

### 2.3. Extraction of Local Orientations

**Figure 9:**
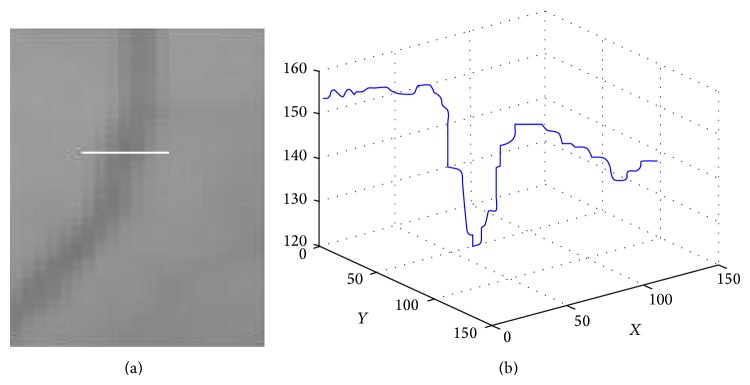
Example of cross sectional profile of blood vessel from gray scale 2D image (the gray intensities are plotted in a 3D view. The *x*, *y* axis is the position of the pixel in the 2D plane of the image, whereas the *z*-axis is the gray value or intensity of the pixel).

The proposed model assumes that the intensity profile of the vessels in the cross section is Gaussian (Figure 9). This is a common assumption that it is employed in numerous algorithms [28, 35, 49]. It is also commonly assumed that the intensity does not change much along vessels [49–51]. Recently, the Hessian matrix could be used to describe the local shape characteristics and orientation for elongated structures [35, 52]. The eigenvalues of this matrix, when the gradient is weak, express the local variation of the intensity in the direction of the associated eigenvectors. Subsequently, we assume that we want to characterize the dark vessels (low intensity) on a white background (high intensity).

**Figure 10:**
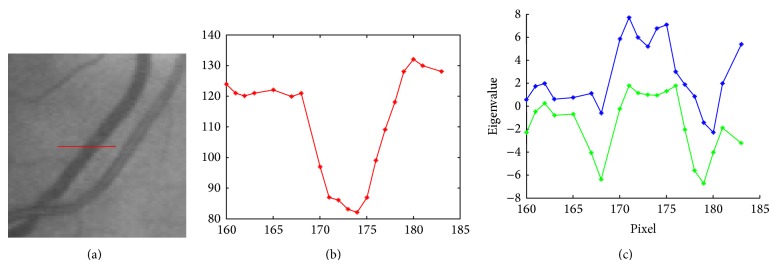
Eigenvalue analysis. (a) vessel cross section; (b) intensity distribution (*σ* = 4.55) vessel cross section; (c) corresponding eigenvalues.

Let us denote *λ*_1_ and *λ*_2_ as the eigenvalues of the Hessian matrix with *λ*_1_ ≥ *λ*_2_ and $\vec{v_{1}}$, $\vec{v_{2}}$ being their associated eigenvectors (Figure 10). For a linear model with a Gaussian cross section, the vessel direction is defined by the eigenvector with the smallest eigenvalue at the center of the vessel, but it is less determined at the contours because both eigenvalues of the Hessian matrix are zero.

To summarize, for an ideal linear structure in a 2D image,

(16) $$\begin{array}{c} \left|\lambda_{2}\right|\approx 0,\\ \left|\lambda_{1}\right|>\left|\lambda_{2}\right|. \end{array}$$

**Figure 11:**
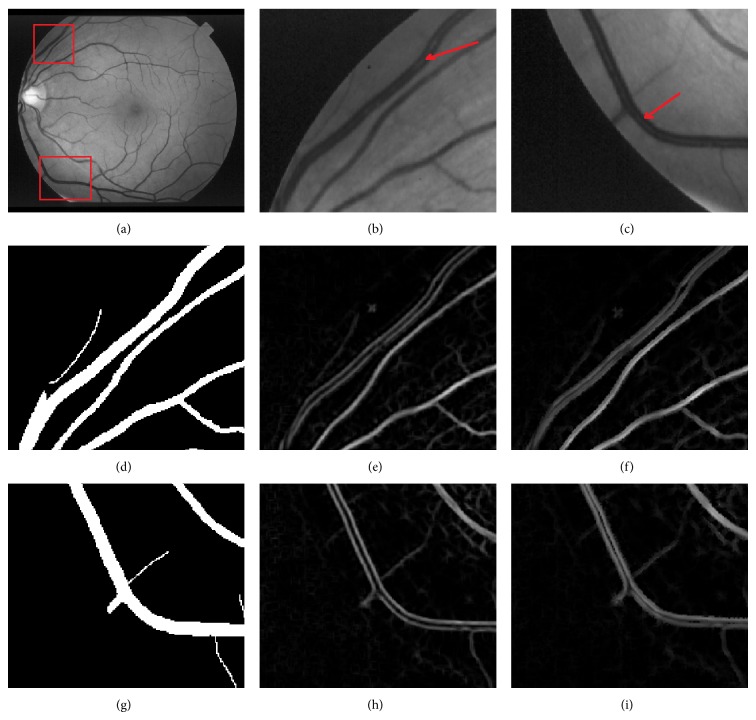
Retinal blood vessel detection. (a, b, and c) original images [33]; (d–g, e–h, and f–i) subimage of hand labeled image, vessel detection based Hessian eigenvalue analysis, and improved vessel detection with gradient magnitude.

In retinal images, some large vessels may have a white line in their center and some elongated and disjoint spots (Figures 11(a), 11(b), and 11(c)); accordingly, the vessels do not invalidate the Gaussian profile assumption. So, such lines are usually lost after the preselection of vessel pixels using the Hessian eigenvalue analysis and classified as background pixels. Therefore, the responses of the gradient magnitude are a task which is of particular importance in improving the detection vessels (Figure 11). The experimental results are demonstrated in Figure 11, which shows hand labeled “truth” images, and segmented images obtained, respectively, by the Hessian eigenvalue analysis and the gradient magnitude. From these results we can deduce that responses based on the gradient magnitude can availably detect white lines as vessel pixels an removes some noise spots.

## 3. Results

**Figure 12:**
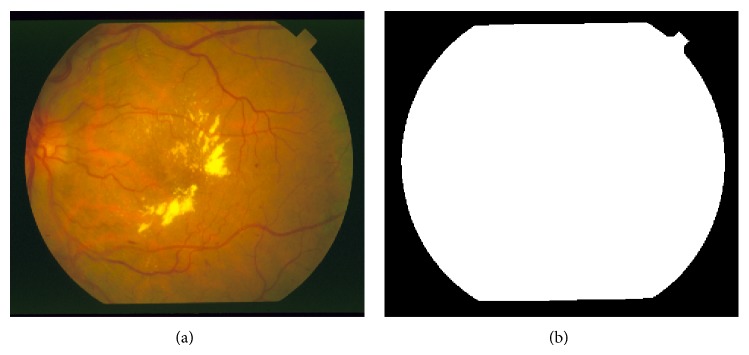
Binary mask of STARE project retinal image [33].

In this section, the proposed method has been evaluated on two publicly available retinal image databases, the STARE database [33] and the DRIVE database [25]. The STARE dataset contains twenty fundus colour retinal images, ten of which are from healthy ocular fundi and the other ten are from unhealthy ones. These images are captured by a Topcon TRV-50 fundus camera at a 35 Field Of View (FOV), which have digitized with a 24-bit gray-scale resolution and a size of 700 × 605 pixels. The dataset provides two sets of standard hand-labeled segmentations, which are manually segmented by two eye specialists. We create for this dataset a binary mask of the gray channel of the image using a simple threshold technique (Figure 12). We adapt the first eye specialist hand labelled as the ground truth to evaluate our vessel detection technique. The DRIVE dataset consists of 40 fundus ocular images, which have been divided into a training set and a test set by the authors of the database. These images are captured by the Canon CR5 camera at 45 FOV, which have been digitized at 24 bits with a resolution of 565 × 584 pixels. The dataset also gives two sets of standard hand-labeled segmentations by two human experts as a 9-ground truth.

**Figure 13:**
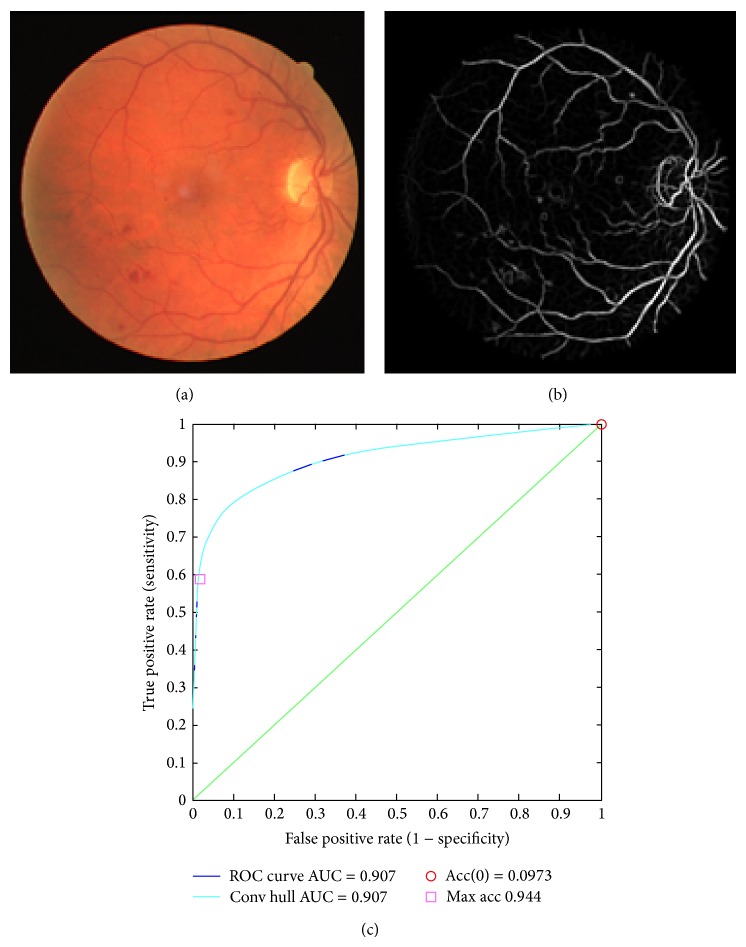
ROC curve of retinal image (06_test.tif) downloaded from DRIVE dataset [34]; (a) original image; (b) segmented image; (c) Roc curve.

The first expert hand labelled segmentation has been adapted as a ground truth to evaluate segmentation techniques on both STARE and DRIVE datasets. It is a common practice to evaluate the performance of retinal vessel segmentation algorithms using receiver operating characteristic (ROC) curves [25, 35]. An ROC curve plots the fraction of pixels correctly classified as vessels, namely, the true positive ra te (TPR), versus the fraction of pixels wrongly classified as vessels, namely, the false positive rate (FPR), by varying the rounding threshold *T* from 0 to 1 (Figure 13). The closer the curve approaches the top left corner, the better the performance of the system. In order to facilitate the comparison with other retinal vessel detection algorithms, we have selected the value of the area under the curve (AUC), which is 1 for an ideal system.

To measure the performance of the proposed enhancement filter, we ran our multiscale analysis filter with the following set of parameters:

- *r*_min⁡_, *r*_max⁡_, *s*, and the minimal and maximal radii used in this application are *r*_min⁡_ = 1.25 and *r*_max⁡_ = 7, discretized using *s* = 4 scales;
- the parameter *γ* set to one to indicate no scale is preferred;
- the value *k* is a constant threshold on the norm of gradient on the image;
- *N* is the number of iterations for the anisotropic diffusion filter.

**Figure 14:**
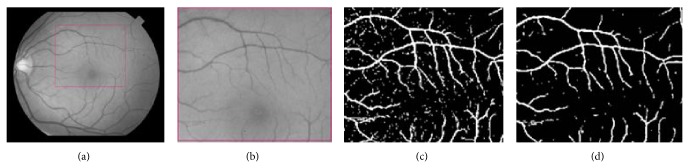
Effect of anisotropic diffusion. (a) Green channel of the original image downloaded from the STARE project dataset [33]. (b) Subimage of the original image, rescaled for better visualization, (c) segmentation without anisotropic diffusion, and (d) segmentation with anisotropic diffusion, *k* = 1.25, *β* = 0.05, and *N* = 30.

The computing time of our algorithm for an image of the STARE database is about 64 seconds, including anisotropic diffusion filtering, and about the same time for the DRIVE database. The implementation of the filter has been done in MATLAB, on a personal computer with a 2.13 Intel Core Duo processor and 4 GB of memory. In the first experiment, we apply a preprocessing task such as filtering data with an anisotropic diffusion version, cited above, in order to remove or at least reduce noise. The DAD filter denoises the original image by preserving edges and details. To show that the segmentation works better with anisotropic diffusion, Figure 14 presents a segmentation result before and after the application of the anisotropic diffusion scheme. In this figure, we show the improvements provided by the DAD model, which tends to remove noise effects and, unfortunately, smaller objects. So, it preserves efficiently the vessels while making the background more homogeneous.

**Figure 15:**
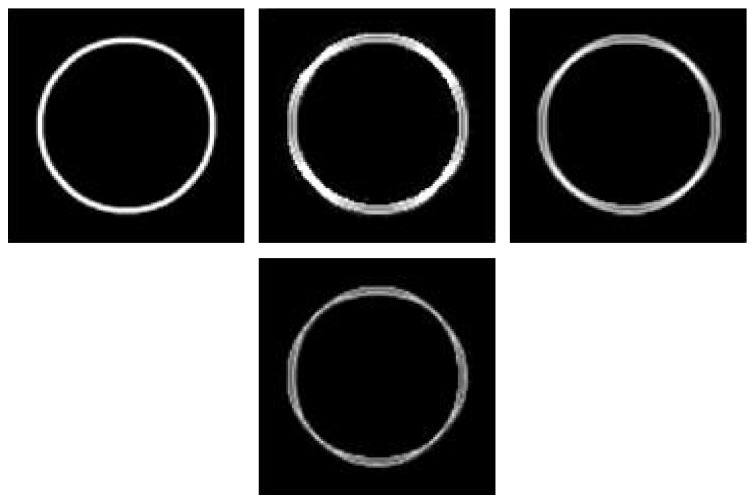
Original synthetic image, maximum response, average response, and minimum response *σ* ∈ {0.25,0.5,1, 2,4} (left to right-top to bottom).

On the other hand, for computing the response, it is possible to retain the mean of the two calculated values (the gradient of the two points located at an equal distance from the current point), like in the 3D case proposed by [36], or the minimal calculated value in the 2D case [37]. We prefer retaining the maximum of these two values.  Figure 15 shows a synthetic image which consists of 100 × 100 pixels with an 8-bit resolution. We have chosen this image because it contains an object close to the vessel form. The latter figure shows the segmentation results by maximum, average, and minimum response functions. We note that for the case of minimum or average responses, the ring is not completely detected like in the original image, since we can see it has been missing pixels belonging to the edges, in contrast to maximum case where the extraction of the ring is complete.  Table 4 presents the AUC calculated with our method for the test set of the STARE database, using the green channel images. As given in the table, the experimental results show that the maximum model (AUC = 0.9445) performs much better than the average (AUC = 0.9329) or minimum model (AUC = 0.9053).

**Figure 16:**
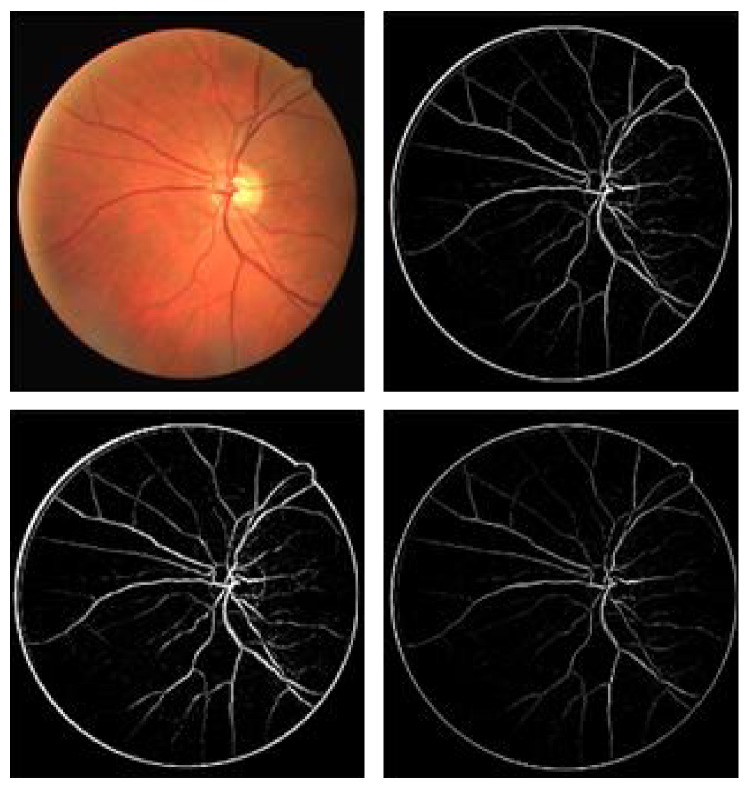
Real angiography image downloaded from DRIVE dataset [34], average response, maximum response, and minimum response (left to right-top to bottom).

Figure 16 presents the obtained response image of a real retinal image, where four scales have been used for radii of vessels ranging from 1.25 to 7: {1.25,2.22,4, 7}. This figure shows that small and large vessels can be better distinguished in the maximum case than the minimum or average ones.

**Figure 17:**
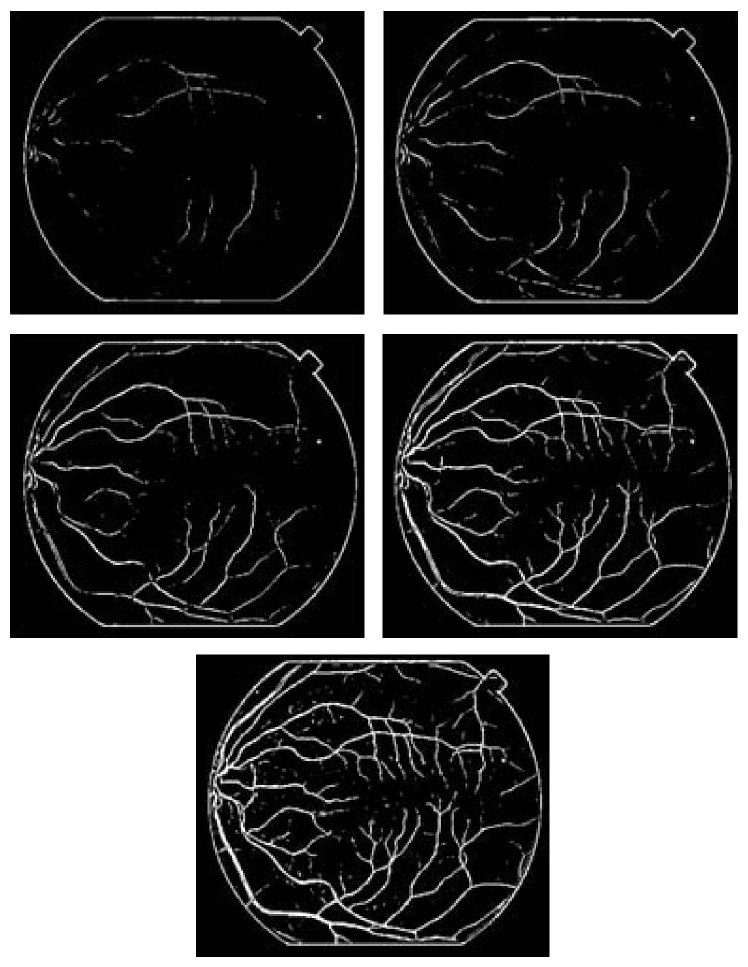
Different responses for different scales of Figure 14(a) (top to bottom); the first four images show the vesselness obtained at increasing scales. The last image is the result after the scale selection procedure (normalized image).

**Figure 18:**
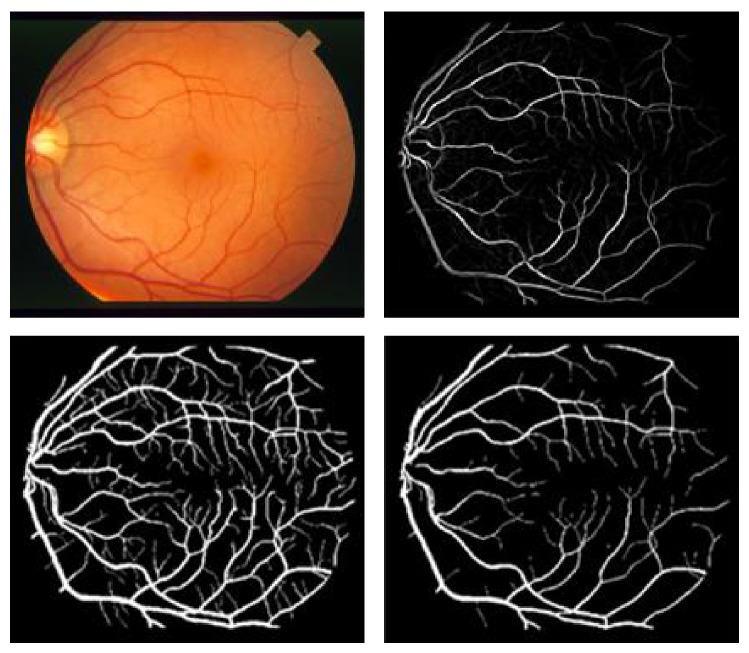
An image of a retina [35], the segmented image, and the hand labeled “truth” images (im0077.vk and im0077.ah) (left to right-top to bottom) [33].

Although the contrast is not very high in the original figure (Figure 14(a)), the method detects most vessels, over a large size range. For example, in Figure 17, an image of the retinal tree vasculature is presented, where different responses recorded at increasing scales are represented. The last image shows a quite good performance of the vessel subtraction. Yet Figure 18 proves that it is possible to design a system that approaches the performance of human observers.

In order to evaluate the suggested method, experiment results of the 20-image sets of the STARE database are shown in Table 5. In Table 6, our method is compared to the most recent methods in terms of TPR, FPR, and maximum accuracy average (MAA) where the maximal accuracy indicates how to extract a binary image that matches the vessel images to a high degree. The accuracy is estimated by the ratio of the sum of the number of correctly classified foreground and background pixels, divided by the total number of pixels in the image. In this latest table, the performance measures of Staal et al. [25], Zhang et al. [14], Mendonça and Campilho [21], Chaudhuri et al. [13], Martinez-Perez et al. [45], and Hoover et al. [35] have been reported by their original papers. In addition, these performance results are the average values for the whole set of 20 images, except the method of Staal [25] which used 19 out of 20 images of the STARE images, among which ten were healthy and nine were unhealthy. Table 5 presents our results on all 20 images in the STARE database, estimated using the hand-labeled images set of the first human expert designated as a ground truth. The estimated experimental results are the average TPR = 0.6145 corresponding to an FPR of around 0.0162 and a maximum average accuracy MAA = 0.9402. The results show that our method has a competitive maximum average accuracy value where it performs better than the matched filter [13] and remains close to the others.

The results of the proposed method are also compared with those on twenty images from the DRIVE database, and the result is depicted in Table 7. The hand-labeled images by the first human expert have been used as ground truth. The experimental results show an MAA around of 0.9155. Also, we have compared the performance of the suggested technique with the sensitivities and specificities of the methods cited in Table 7. It has been found that for the DRIVE database the method has provided a sensitivity of 0.5879 and a specificity of 0.0166. We have shown that the proposed method performs well with a lower specificity even in the presence of lesions in the abnormal images.

## 4. Conclusion

The purpose of this work is to detect linear structures in real retinal images in order to help the interpretation of the vascular network. We put forward to combining an anisotropic diffusion filter to reduce the image noise with a multiscale response based on the eigenvectors of the Hessian matrix and on the gradient information to extract vessels from retinal images. The main advantage of this technique is its ability to extract large and fine vessels at various image resolutions. Furthermore, the directional anisotropic diffusion plays a vital role in denoising images and in decreasing the difficulty of vessel extraction especially for thin vessels. Our first results show the robustness of the method against noise as well as its applicability to detect blood vessels. The MAA is used as a performance measure, and the values achieved with our algorithm are competitive compared to the existing methods. Therefore, from the experimental results, it can be seen that the number of classified pixels has been slightly lower compared to the other methods using the same database mainly due to the weakness of blood vessels, causing missing vessels, and also because of lesions, resulting in a detection error. Also, the retinal images suffer from nonuniform illumination and have a poor contrast. Thus, to avoid wrong classified pixels or miss classified ones, caused by an occasional false measurement, this system can very well be improved in the future with adding, for instance, some postprocessing tasks to reach more accurate measurement for blood vessels.

## Figures and Tables

**Table 1:** Parameters and results of different filters for vessel image.

Filter	*N*	*k*	β	σ	*dt*	Neig.	PSNR (dB)	MSE
GF	—	—	—	2	—	9 × 9	37.7717	10.8620
MF	—	—	—	—	—	5 × 5	38.6364	8.9011
PM	13	7	—	—	0.15	—	39.6735	7.0103
**DAD**	**50**	**7**	**0.05**	**0.8**	**0.05**	—	**40.4337**	**5.8845**

**Table 2:** Parameters and results of different filters for phantom image.

Filter	*N*	*k*	β	σ	*dt*	Neig.	PSNR (dB)	MSE
GF	—	—	—	2	—	5 × 5	18.8731	842.8924
MF	—	—	—	—	—	5 × 5	20.2437	614.7677
PM	20	3	—	—	0.15	—	20.8821	530.7294
**DAD**	**75**	**2**	**0.05**	**0.8**	**0.05**	—	**20.9045**	**527.9932**

**Table 3:** Parameters and results of different filters for Lena image.

Filter	*N*	*k*	β	σ	*dt*	Neig.	PSNR (dB)	MSE
GF	—	—	—	2	—	5 × 5	31.4598	46.4621
MF	—	—	—	—	—	5 × 5	29.14504	79.1734
PM	10	7	—	—	0.15	—	32.9911	32.6562
**DAD**	**20**	**7**	**0.05**	**0.8**	**0.05**	—	**33.3515**	**30.0557**

**Table 4:** STARE project database [33].

	Mean	Min	Max
AUC	0.9329	0.9053	**0.9445**

**Table 5:** ROC curve analysis of STARE project database [33].

Number	MAA	TPR	FPR
1	0.9014	0.5537	0.0398
2	0.8740	0.1178	0.0045
3	0.9168	0.3819	0.0119
4	0.9286	0.5525	0.0135
5	0.9240	0.5678	0.0218
6	0.9414	0.5128	0.0139
7	0.9672	0.7626	0.0141
8	0.9683	0.7534	0.0149
9	0.9652	0.7366	0.0123
10	0.9420	0.6171	0.0182
11	0.9503	0.6379	0.0133
12	0.9655	0.7694	0.0105
13	0.9864	0.6992	0.0180
14	0.9480	0.6899	0.0162
15	0.9487	0.6882	0.0207
16	0.9226	0.6788	0.0215
17	0.9499	0.7099	0.0168
18	0.9484	0.6812	0.0102
19	0.9585	0.6058	0.0114
20	0.9345	0.6000	0.0172

	Av.MAA	Av.TPR	Av.FPR
	0.9402	0.6145	0.0162

**Table 6:** Comparison of vessel segmentation results on STARE project database [33].

Method	MAA	TPR	FPR
2nd human observer	0.9354	0.8949	0.0610
Hoover [33, 35]	0.9267	0.6751	0.0433
Mendonça (green) [21]	0.9440	0.6996	0.0270
Staal [25]	0.9516	0.6970	0.0190
Soares [44]	0.9480	0.7165	0.0252
Matched filter [13]	0.9384	0.6134	0.0245
Martinez-Perez [45]	0.9410	0.7506	0.0431
MF-FDOG [14]	0.9484	0.7177	0.0247
**Proposed method **	**0.9402 **	**0.6145**	** 0.0162 **

**Table 7:** Comparison of vessel segmentation results on DRIVE database [34].

Method	MAA	TPR	FPR
2nd human observer [34]	0.9473	0.7761	0.0275
Martinez-Perez [45]	0.9344	0.7246	0.0345
Staal [25, 34]	0.9442	0.7194	0.0227
Mendonça [21]	0.9452	0.7344	0.0236
Matched filter [13]	0.9284	0.6168	0.0259
Niemeijer [34, 46]	0.9417	0.6898	0.0304
**Proposed method**	**0.9155**	**0.5879**	** 0.0166**
